# Investiture by the King

**Published:** 1921-03-12

**Authors:** 


### Investiture by the King.

His Majesty the King held an Investiture at Buckingham Palace on March 8, when he conferred decorations of the Royal Red Cross as follows:—

*Received a Bar*: Matrons Jane Dods, Helena Hartigan, and Dorothea Taylor, and Sister Gertrude Allen, all of Queen Alexandra's Imperial Military Nursing Service; Matrons Millicent Acton and Annie Earle, Territorial Force Nursing Service.

*Members*: Matron Agnes Wilson, Sisters Gladys Howe, Winifred Jones, and Joanna Murphy, all of Q.A.I.M.N.S.; Lady Superintendent Isabel Lloyd, Q.A.I.M.N.S. (India); Matrons Eleanor Jones and Mary Rae, Sisters Margaret Greig, Gwenllian Roberts, and Annie Sayer, all of Q.A.I.M.N.S. Reserve; Matrons Agnes Brooks, Laetitia Clark, Annie Kirkham, and Mary Munro, and Sisters Hope Dibben, Miss Emma Bramwell, and Miss Hannah Newton, all of Civil and War Hospitals.

Mrs. Lilian Doughty-Wylie, who was invested with the insignia of an Officer of the Order of the British Empire, also received the Royal Red Cross.

